# Atrial Fibrillation Termination Success During Ablation: Insights From Pooled Clinical Studies

**DOI:** 10.31083/RCM33419

**Published:** 2025-07-30

**Authors:** Changjian He, Wenchang Zhang, Feng Li, Huaiqiang Wang, Xiongyi Han, Zihan Zhao, Guojie Ye, Tengfei Liu, Da Zhang, Haiyan Liu, Jie Liu, Jingning Zhao, Chunhua Ding

**Affiliations:** ^1^Cardiac Department, Aerospace Center Hospital (Peking University Aerospace School of Clinical Medicine), 100049 Beijing, China; ^2^Department of Cardiology, The Second Affiliated Hospital of Soochow University, 215004 Suzhou, Jiangsu, China

**Keywords:** atrial fibrillation, catheter ablation, acute termination, meta-analysis

## Abstract

**Background:**

The optimal endpoint for ablation in persistent atrial fibrillation (pers-AF) remains unclear. This study aimed to systematically evaluate the prognostic value of acute AF termination in predicting the recurrence of arrhythmias.

**Methods:**

A systematic search of the PubMed, Cochrane Library, Web of Science, and Embase databases was conducted from inception to July 2023. Only studies with reports of acute termination for pers-AF and its predictive role in arrhythmia recurrence were included. Subgroup analysis was performed to identify potential confounders for the effect of AF termination.

**Results:**

A total of 22 studies were included in the meta-analysis. The pooled analysis indicated that acute termination of AF is significantly associated with an increased long-term success rate (relative risk (RR), 1.53; 95% CI, 1.41–1.66; *p* < 0.001; I^2^ = 35.4%). Moreover, subgroup analysis revealed that patients with an AF duration >12 months (RR, 1.92; 95% CI, 1.57–2.35; *p* < 0.001), aged >60 years (RR, 1.92; 95% CI, 1.60–2.31; *p* < 0.001) may derive benefits from AF termination during ablation. Interestingly, a significant interaction was observed in the study design subgroup, where multi-center studies showed a success rate of RR, 1.31 (95% CI, 1.14–1.50; *p* < 0.001), while single-center studies exhibited a higher success rate of RR, 1.65 (95% CI, 1.49–1.82; *p* < 0.001), with an interaction *p*-value of 0.008. Importantly, acute termination of AF did not significantly increase procedural complications (RR, 1.19; 95% CI, 0.59–2.39; *p* = 0.627; I^2^ = 0.0%).

**Conclusions:**

Our study suggests that AF acute termination during ablation for pers-AF provides a better long-term clinical outcome.

**The PROSPERO Registration:**

CRD42023431015, https://www.crd.york.ac.uk/PROSPERO/view/CRD42023431015.

## 1. Introduction

Atrial fibrillation (AF) is a common type of arrhythmia encountered in clinical practice, and ablation therapy is recommended as the first-line treatment, especially for paroxysmal AF (PAF) [1]. Nevertheless, the optimal endpoint for catheter ablation in patients with persistent atrial fibrillation (pers-AF) remains undefined. Current evidence suggests that atrial arrhythmia non-inducibility should not be utilized as a procedural endpoint, as its clinical relevance and predictive value for long-term outcomes have not been substantiated [2, 3]. While some studies hypothesize that acute termination of AF may correlate with improved clinical outcomes, this association remains controversial, and using acute termination of AF as the end point of ablation in case of pers-AF is challenging and typically requires extensive left atrial ablation [4, 5]. The stepwise ablation for pers-AF typically involves pulmonary vein isolation (PVI) followed by additional substrate modification techniques, such as linear ablation or complex fractionated atrial electrogram (CFAE) ablation [6, 7]. This comprehensive strategy combines PVI with substrate modification, and has a higher termination rate for AF compared to PVI-only strategies or other ablation strategies applied alone [8, 9]. Nonetheless, despite the higher termination rate of cardiac arrhythmias with this comprehensive approach, the significant recurrence rate of organized arrhythmias following extensive and delicate radiofrequency ablation makes this method controversial. Therefore, identifying the optimal ablation strategy for pers-AF continues to be a challenge [4, 10, 11].

The acute termination of AF during ablation suggests the successful elimination of the key drivers or substrates for the maintenance of pers-AF. However, if AF persists after ablation, it indicates that the key drivers or substrate of AF may still be present, potentially increasing the likelihood of AF recurrence post-ablation. A previous study has shown that a PVI-only strategy yielded similar results to extensive ablation strategies for pers-AF [12]. However, the ablation effect of pers-AF is still not optimal compared with paroxysmal AF, suggesting that the potential trigger or drivers beyond pulmonary vein (PV) might be neglected and underestimated [13]. In addition, accumulated studies showed conflicting findings regarding the clinical significance of acute termination of AF [4, 14, 15, 16]. Therefore, AF termination as a catheter ablation endpoint is still in debate.

This study aims to systematically evaluate the role of AF acute termination in predicting arrhythmia recurrence. Since acute termination is not a commonly designated programmed end point, only studies that explicitly reported acute termination and evaluated its predictive role in arrhythmia recurrence were included.

## 2. Methods

### 2.1 Search Strategy

We performed a systematic review with a protocol registered on PROSPERO (CRD42023431015, https://www.crd.york.ac.uk/PROSPERO/view/CRD42023431015) using the PRISMA guidelines.

Our research, conducted from databases inception to July 2023, included PubMed, Cochrane Library, Web of Science, and Embase databases. All stages of the review process, including study selection, data extraction, and quality assessment, were independently conducted by two reviewers (CJH and FL) to minimize bias. The search terms used were “termination”, “ablation”, and “atrial fibrillation”. Furthermore, we manually screened the reference lists of review articles to identify any eligible publications that may not have been captured in the initial search.

### 2.2 Study Design

Clinical studies were included based on the following inclusion criteria: (1) Randomized controlled trials, prospective observational studies, and retrospective observational studies; (2) Studies that compared the outcomes of AF termination during the procedure, including long-term freedom from AF/atrial tachycardia (AT)/atrial flutter (AFL) and acute AF termination rate. Acute termination of AF was defined as conversion to sinus rhythm (SR) or a transient intermediate rhythm (AT/AFL) that was subsequently mapped and ablated to achieve SR. Patients who required cardioversion to restore SR were non-termination group. Long-term success rate refers to the success rate of patients being free from AF/AT/AFL after a follow-up period of more than 12 months. (3) Eligibility for inclusion was restricted to full-text studies published in English in peer-reviewed journals; (4) When multiple publications of the same trial or cohort were available, studies with the most comprehensive data were prioritized for inclusion. The exclusion criteria included case reports, letters, single-arm studies, and animal studies. The research was independently carried out by CJH and FL Any disagreements about eligibility were settled through discussion with a third reviewer (CHD). The main outcome was long-term freedom from AF/AFL/AT while the secondary outcomes assessed operative complication rates.

### 2.3 Data Extraction and Quality Assessment

In each eligible study, data extraction was independently conducted by two researchers (CJH and FL), with any discrepancies resolved through discussion involving a third researcher (CHD). For randomized controlled trials, the Cochrane Risk of Bias tool was utilized for assessment. Observational studies underwent evaluation using the Newcastle-Ottawa Quality Assessment Scale (NOS). Furthermore, we conducted an assessment for potential publication bias using Egger’s test.

### 2.4 Statistical Analysis

Statistical analyses were performed using Stata version 16.0 (StataCorp LP, College Station, TX, USA). A two-tailed *p* < 0.05 was considered statistically significant. Categorical variables were expressed as frequencies and percentages, while continuous variables were summarized as means ± standard deviations or as medians with interquartile ranges (IQRs), depending on their distribution.

We used I^2^ statistics to evaluate the heterogeneity. An I^2^ value exceeding 50% warranted the use of a random-effects model, while a fixed-effects model was applied when the I^2^ value was below this threshold. Additionally, a sensitivity analysis was conducted to assess the influence of individual studies on the overall risk estimate, achieved by systematically excluding one study at a time, particularly in the presence of significant heterogeneity. Egger’s tests were employed to evaluate potential publication bias.

Additionally, we conducted subgroup analyses to identify sources of heterogeneity and potential confounders for AF ablation outcomes between the AF termination and non-termination groups. Several potential factors, including study design, follow-up time, sample size, gender distribution, age cutoff, AF duration, presence of diabetes mellitus (DM), hypertension (HT), left atrial diameter (LAD), and left ventricular ejection fraction (LVEF) were considered. We categorized the study design as single-center or multicenter. Follow-up time and AF duration were stratified as >12 months and ≤12 months. Sample size and age cutoff were divided using cutoff values of 50 and 60, respectively. Gender, DM, and HT proportions were categorized based on cutoff values of 80%, 10%, and 50%, respectively. LAD and LVEF were stratified using cutoff values of 40 mm and 55%, respectively.

## 3. Results

### 3.1 Study Selection and Quality Assessment

**Fig. 1.**
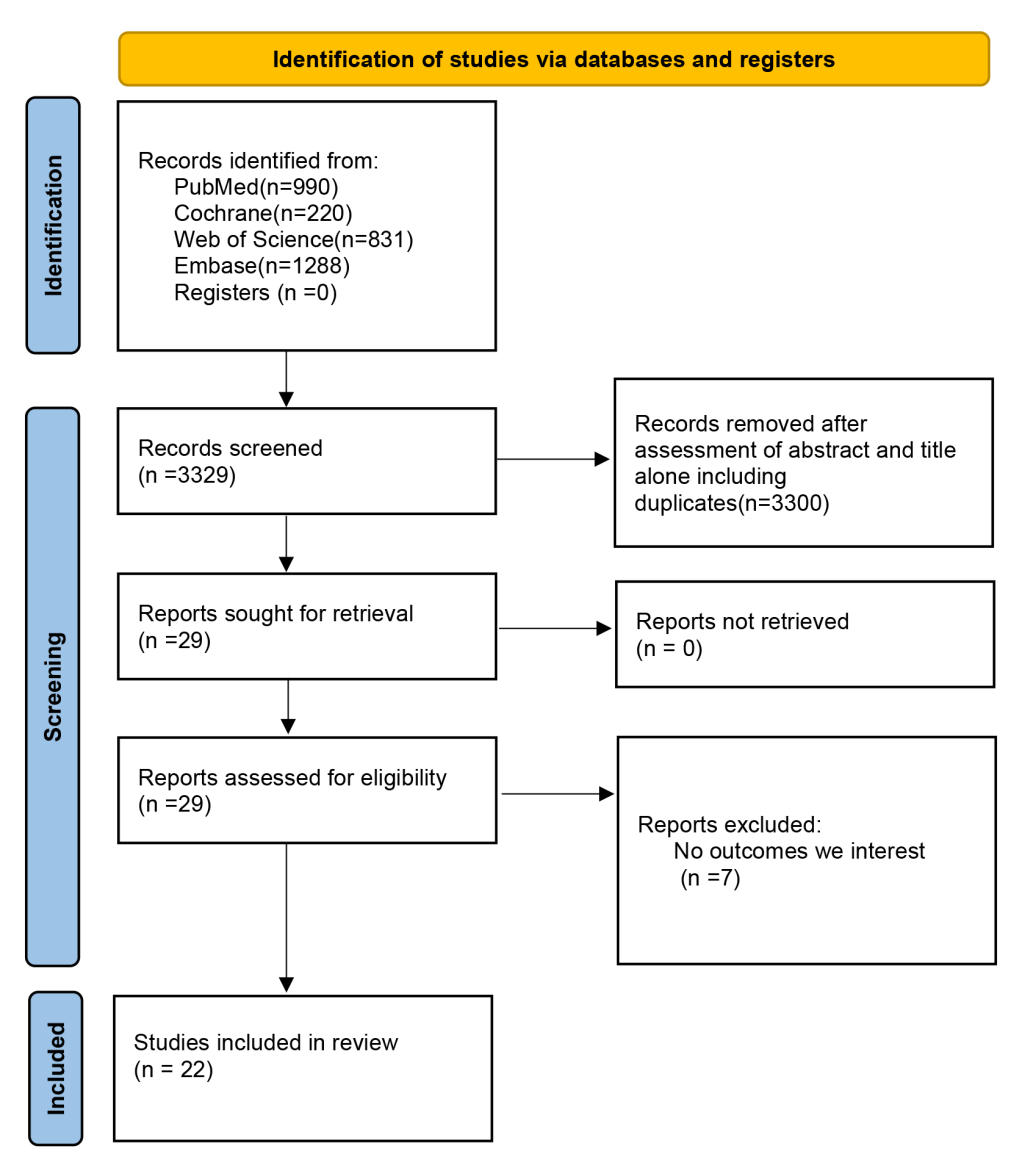
**The research selection flowchart**.

The process for selecting studies is illustrated in the flowchart presented in Fig. 1. Two investigators independently screened the abstracts, resulting in 22 studies that met our inclusion criteria. These included 2 multicenter study and 20 single-center studies, involving a total of 3080 patients with AF, with 1655 in the AF termination group and 1425 in the AF non-termination group. The characteristics of these studies can be found in Table 1 (Ref. [4, 8, 16, 17, 18, 19, 20, 21, 22, 23, 24, 25, 26, 27, 28, 29, 30, 31, 32, 33, 34, 35]) (Table 1. continued was shown in the **Supplementary Material**. It provides information on follow-up duration, follow-up strategy and the application of antiarrhythmic drugs). The baseline characteristics of the included populations, both overall and stratified by acute termination status are in the **Supplementary Table 1**. Overall, patients in the termination group were younger (58.3 ± 9.1 years vs. 60.2 ± 10.4 years in the non-termination group) and had smaller LAD (43.2 ± 5.0 mm vs. 46.1 ± 6.2 mm). Male gender predominated in both groups (76.5% termination vs. 78.2% non-termination). Comorbidities such as hypertension (52% vs. 58%) and diabetes (18% vs. 22%) were slightly less prevalent in the termination group. Among the 22 studies included in the meta-analysis, 1 was a randomized controlled trial (RCT), 5 were prospective observational studies, and 16 were retrospective observational studies. The quality of the eligible studies varied from moderate to good, as shown in Table 2 (Ref. [4]) and Table 3 (Ref. [8, 16, 17, 18, 19, 20, 21, 22, 23, 24, 25, 26, 27, 28, 29, 30, 31, 32, 33, 34, 35]). Radiofrequency ablation was used in all procedures. The ablation strategies varied, however, the primary approach was a “stepwise” strategy, which involved PVI combined with linear ablation, CFAE, driver ablation, and other techniques. In the included studies, freedom from AF was defined as the absence of any documented recurrence of AF/AT/AFL.

**Table 1.** **The demographic and clinical characteristics**.

First author, year, reference	Study design	Center	Sample size		Gender (male%)		Age		LAD (mm)		Ablation Technique	Type of AF Termination
			Term	Non-Term	Term	Non-Term	Term	Non-Term	Term	Non-Term		
Li-2023 [4]	Prospective study (RCT)	multicenter center	216	234	NA	NA	NA	NA	NA	NA	PVI+Linear ablation/VOM ethanol infusion/Driver ablation	SR/AT/AFL
Riku-2023 [17]	retrospective	single-center	12	24	9 (75)	16 (67)	64.8 ± 8.8	63.3 ± 14.0	44.9 ± 3.0	42.4 ± 6.8	PVI+Linear ablation/Driver ablation	SR/AFL
Park-1-2022 [18]	retrospective	single-center	86	44	68 (79.1)	40 (90.9)	60.8 ± 10.0	57.6 ± 9.3	41.5 ± 5.1	46.5 ± 4.9	PVI+CFAE	SR/AT/AFL
Honarbakhsh-2022 [19]	retrospective	single-center	54	14	39 (76.5)	8 (57.1)	61.4 ± 8.2	59.6 ± 11.8	41 ± 4	39 ± 4	PVI+Drivers ablation	SR/AT
Wang-2016 [16]	retrospective	single-center	76	35	61 (77.6)	28 (80)	56 ± 10	56 ± 12	NA	NA	PVI+Drivers ablation/CFAE	SR
Buttu-2016 [20]	retrospective	single-center	21	9	19 (90.5)	9 (100)	61 ± 8	61 ± 4	NA	NA	PVI+Linear ablation/CFAE	SR/AT
Yuen-2015 [21]	retrospective	single-center	110	26	92 (83.6)	21 (80.8)	56.8 ± 11.1	59.9 ± 12.1	43.1 ± 5.4	47.5 ± 6.7	PVI, LA CFAE ablation, RA CFAE ablation, and linear ablation	SR
Wu-2014 [22]	retrospective	single-center	72	45	61 (81.3)	33 (73)	53.9 ± 10.5	53.1 ± 10.0	40.7 ± 4.3	43.2 ± 5.1	pure linear ablation without a CPVI	SR
Faustino-2014 [23]	prospective	single-center	135	70	84 (62.2)	47 (70)	61.6 ± 7.3	63.8 ± 6.9	44.4 ± 3.4	46.3 ± 3.9	PVI+CFAE	SR
Chen-2013 [24]	retrospective	single-center	48	49	40 (83.3)	40 (81.6)	58 ± 11	55 ± 12	43.5 ± 5.2	44.2 ± 6.7	PVI+LA/RA CFAE/Linear ablation	SR
Zhou-2013 [25]	retrospective	single-center	125	75	98 (78.4)	58 (77.3)	56.5 ± 11.4	58.6 ± 9.8	45.3 ± 5.8	47.0 ± 6.8	PVI, CFAE, Linear ablation	SR/AT
Combes-2013 [26]	prospective	single-center	18	22	14 (77)	19 (86)	58.7 ± 13.5	61.1 ± 8.6	NA	NA	PVI+Linear ablation/CFAE	SR
Kumagai-2013 [27]	retrospective	single-center	14	56	10 (71.4)	46 (82.1)	62.0 ± 8.0	62.2 ± 8.0	44.7 ± 6.5	46.7 ± 4.8	PVI+Linear ablation/CFAE	SR
Ammar-2013 [28]	retrospective	single-center	62	82	47 (76)	66 (80)	56 ± 13	57 ± 12	46 ± 6	49 ± 6	PVI+Linear ablation/CFAE	SR/AT
Matsuo-2012 [29]	retrospective	single-center	13	27	12 (92.3)	27 (100)	53.3 ± 10.2	53.6 ± 10.4	42.7 ± 3.9	45.7 ± 4.2	PVI+Linear ablation	SR
Heist-2012 [30]	retrospective	single-center	95	48	68 (70)	41 (90)	63 ± 9	62 ± 8	44 ± 7	47 ± 8	PVI+LA/RA CFAE/Linear ablation	SR
Park-2-2012 [31]	prospective	single-center	95	45	81 (85.3)	39 (86.7)	54.8 ± 10.2	55.0 ± 8.9	42.2 ± 5.5	45.5 ± 5.5	PVI+LA/RA CFAE/Linear ablation	SR
Komatsu-2011 [32]	retrospective	single-center	50	31	40 (80)	26 (84)	62 ± 9	65 ± 8	44.7+5.3	47.7+4.4	PVI+CFAE	SR/AT
Lo-2009 [33]	retrospective	single-center	46	39	34 (73.9)	33 (84.6)	55 ± 9	51 ± 9	38 ± 5	44 ± 8	PVI+/Linear ablation/CFAE/non-PV ectopy ablation	SR
O’Neill-2009 [8]	prospective	single-center	130	23	NA	NA	NA	NA	47 ± 8	52 ± 10	PVI+/Linear ablation/CFAE	SR
Wang-2011 [34]	retrospective	single-center	33	94	20 (68.9)	63 (67.0)	56 ± 10	56 ± 12	39.2 ± 4.8	43.7 ± 6.3	PVI+/Linear ablation/CFAE	SR
Kochhäuser-2017 [35]	prospective	multicenter center	143	333	NA	NA	NA	NA	NA	NA	PVI+/CFAE/linear ablationcc	SR

Table 1. continued was shown in the **Supplementary Material**. The table provides information on follow-up duration, follow-up strategy and the application of antiarrhythmic drugs. AT, atrial tachycardia; AF, atrial fibrillation; AFL, atrial flutter; CFAE, complex fractionated atrial electrogram; LA, left atrial; NA, not available; PV, pulmonary vein; PVI, pulmonary vein isolation; VOM, vein of marshall; RCT, randomized controlled trial; RA, right atrial; SR, sinus rhythm; CPVI, circumferential pulmonary vein isolation.

**Table 2.** **Quality assessment for randomized clinical trials according to the Cochrane risk of bias assessment tool**.

First author, year, reference	Random sequence generation (selection bias)	Allocation concealment (selection bias)	Blinding of participants and personnel (performance bias)	Incomplete outcome data (attrition bias)	Selective reporting (reporting bias)	Other bias
Li-2023 [4]	U	U	L	L	L	U

L, low risk of bias; U, uncertain.

**Table 3.** **Quality assessment of enrolled studies according to the Newcastle-Ottawa Quality Assessment Scale (NOS)**.

First author, year, reference	Selection				Comparability	Outcome			Total stars
	Representativeness of the exposed cohort	Selection of the non-exposed cohort	Ascertainment of exposure	Demonstration that outcome of interest was not present at start of study	Comparability of cohorts on the basis of the design or analysis	Assessment of outcome	Was follow-up long enough for outcomes to occur	Adequacy of follow-up of cohorts	
Riku-2023 [17]	★	★	★	★	★★	★	-	★	8
Park-1-2022 [18]	★	★	★	★	★	★	★	★	8
Honarbakhsh-2022 [19]	★	★	★	★	★	★	★	★	8
Wang-2016 [16]	★	★	★	★	★★	★	★	★	9
Buttu-2016 [20]	★	★	★	★	★★	★	★	★	9
Yuen-2015 [21]	★	★	★	★	★	★	★	★	8
Wu-2014 [22]	★	★	★	★	★	★	★	★	8
Faustino-2014 [23]	★	★	★	★	★★	★	★	★	9
Chen-2013 [24]	★	★	★	★	★★	★	★	★	9
Zhou-2013 [25]	★	★	★	★	★	★	★	★	8
Combes-2013 [26]	★	★	★	★	★	★	★	★	8
Kumagai-2013 [27]	★	★	★	★	★★	★	★	★	9
Ammar-2013 [28]	★	★	★	★	★	★	★	★	8
Matsuo-2012 [29]	★	★	★	★	★	★	★	★	8
Heist-2012 [30]	★	★	★	★	★	★	★	★	8
Park-2-2012 [31]	★	★	★	★	★	★	★	★	8
Komatsu-2011 [32]	★	★	★	★	★	★	★	★	8
Lo-2009 [33]	★	★	★	★	★	★	★	★	8
O’Neill-2009 [8]	★	★	★	★	★	★	★	★	8
Wang-2011 [34]	★	★	★	★	★	★	★	★	8
Kochhäuser-2017 [35]	★	★	★	★	★★	★	★	★	9

★: The NOS assessed the quality of literature using a semi-quantitative star system, awarding one star for each criterion met, with a maximum score of nine stars. Higher scores indicate better study quality.

### 3.2 Freedom From AF at Follow-up

**Fig. 2.**
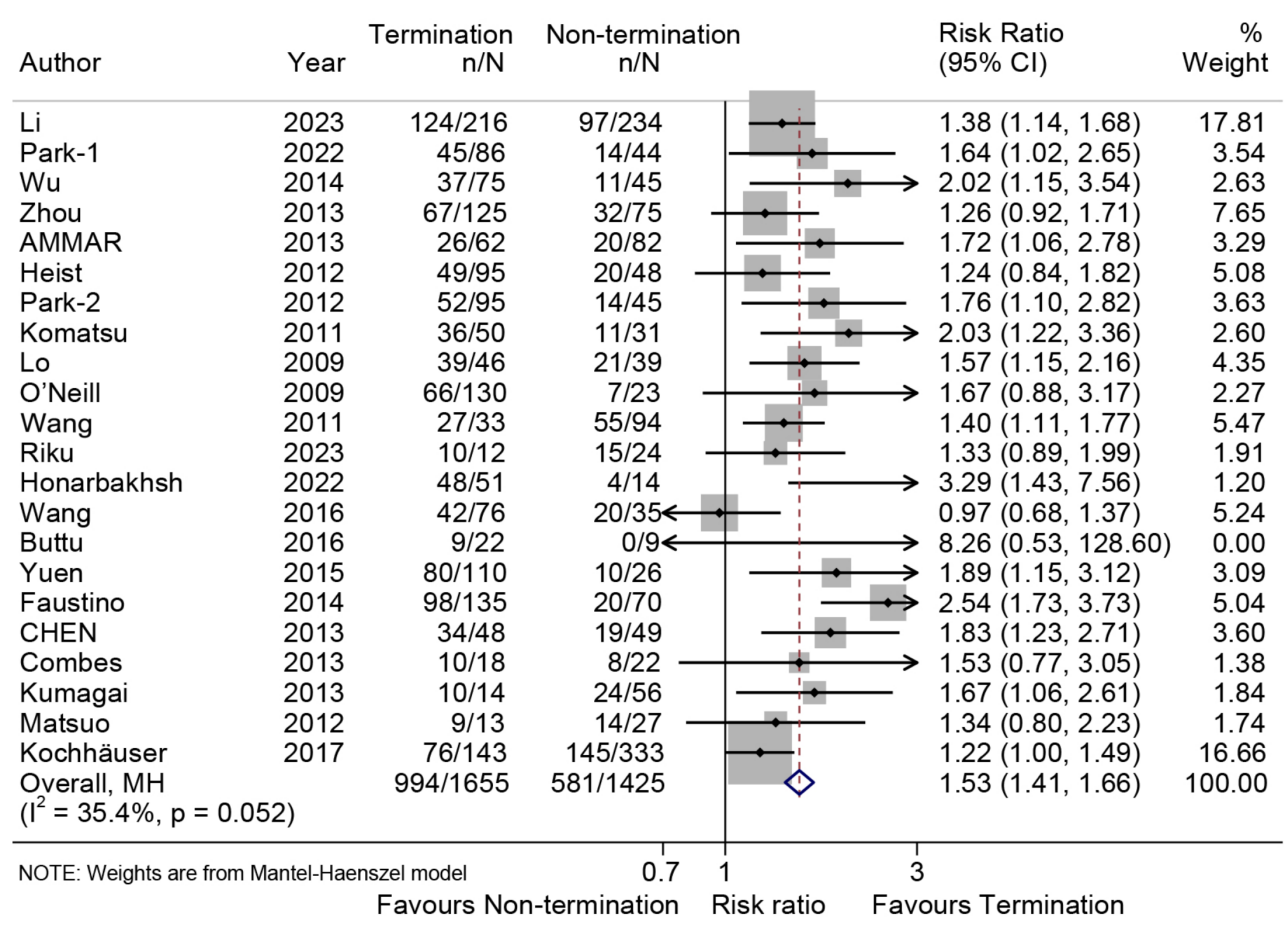
**Forest plot illustrating the long-term freedom from AF/AFL/AT**. The analysis compares the rates of long-term freedom from AF/AFL/AT between the termination group and the non-termination group.

In 22 studies [4, 8, 16, 17, 18, 19, 20, 21, 22, 23, 24, 25, 26, 27, 28, 29, 30, 31, 32, 33, 34, 35],1575 patients were reported to have achieved freedom from AF/AT/AFL, with 63.1% in the AF termination group and 36.9% in the non-termination group. The long-term follow-up showed a significant difference in freedom from AF/AT/AFL between the two groups, with a higher success rate in the termination group (relative risk (RR), 1.53; 95% CI, 1.41–1.66; *p* < 0.001; I^2^ = 35.4%; Fig. 2) using the fixed effect model. And the results of using the random effects model are shown in the **Supplementary Fig. 1**. The results of the two models are consistent, indicating that our results are robust. Sensitivity analysis confirmed the robustness of the findings, with the pooled proportion ranging from 1.53 (95% CI, 1.40–1.68) to 1.65 (95% CI, 1.49–1.82). However, publication bias was detected using Egger’s test (*p* = 0.014), but the adjusted RR with trim-and-fill analysis for publication bias (1.39, 95% CI, 1.24–1.56; **Supplementary Fig. 2**) was similar to the pooled result, indicating the robustness of the findings.

### 3.3 Subgroup Analysis for the Freedom From AF

The study analyzed ten subgroup factors related to freedom from AF and presented the outcomes in **Supplementary Table 2**. Significant differences were observed in all subgroups, consistent with the overall results.

Furthermore, a potentially significant treatment-covariate interaction in the success rate was found in the AF duration subgroup, including AF duration >12 months (RR 1.92; 95% CI, 1.57–2.35; *p* < 0.001) and ≤12 months (RR 1.56; 95% CI, 1.37–1.77; *p* < 0.001), with *p* = 0.084 for interaction. A significant treatment-covariate interaction was identified in the age subgroup analysis, with patients aged >60 years demonstrating a higher success rate (RR 1.92; 95% CI, 1.60–2.31; *p* < 0.001) compared to those aged ≤60 years (RR 1.51; 95% CI, 1.33–1.70; *p* < 0.001), with an interaction *p*-value of 0.030. Similarly, a significant interaction was observed in the study design subgroup, where multi-center studies showed a success rate of RR 1.31 (95% CI, 1.14–1.50; *p* < 0.001), while single-center studies exhibited a higher success rate of RR 1.65 (95% CI, 1.49–1.82; *p* < 0.001), with an interaction *p*-value of 0.008.

### 3.4 Procedure Parameters

**Fig. 3.**
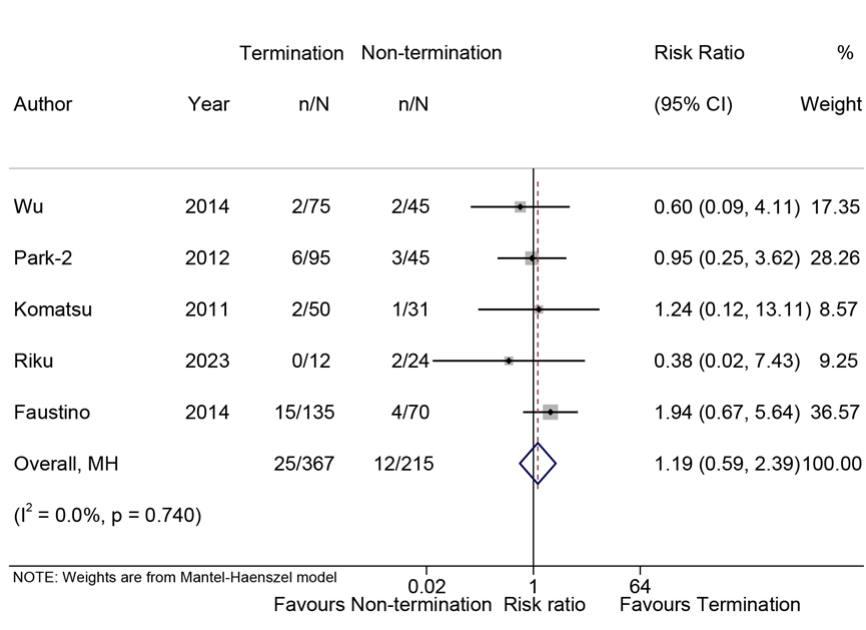
**Forest plot of the complications**. Comparison of the risk of complications in AF termination group and non-termination group.

In five studies [17, 22, 23, 31, 32], operational complications were reported, and the risk of complications in the cohort where AF was terminated demonstrated no significant difference compared to the cohort where AF was not terminated (RR, 1.19; 95% CI, 0.59–2.39; *p* = 0.627; I^2^ = 0.0%; Fig. 3). Sensitivity analysis demonstrated no significant alteration in the pooled proportion, with values ranging from 0.75 (95% CI, 0.29–1.99) to 1.31 (95% CI, 0.62–2.80). Furthermore, Egger’s test did not detect any publication bias (*p* = 0.13).

Total operation time was documented in ten studies [8, 17, 23, 24, 25, 27, 28, 30, 31, 32], total radiofrequency time in eight studies [8, 23, 24, 25, 28, 30, 31, 32], and fluoroscopy time in six studies [23, 24, 25, 28, 31, 32]. The total operation time of the AF termination group was significantly shorter compared to that of the non-termination group, with a standardized mean difference (SMD) of –17.76 (95% CI, –21.65 to –13.87; I^2^ = 42.9%, *p* < 0.001). Additionally, the radiofrequency time was significantly reduced (SMD –10.12; 95% CI, –15.92 to –4.32; I^2^ = 74.0%, *p* = 0.001). There was also a minor decrease in fluoroscopy time for the termination group with an SMD of –7.35; (95% CI, –12.88 to –1.82; I^2^ = 84.2%, *p* = 0.009).

## 4. Discussion

This meta-analysis is the first registered study focused on the acute termination of AF during catheter ablation for pers-AF. Our pooled results suggested that acute termination of AF is associated with a significantly higher long-term success rate. Moreover, subgroup analysis indicated that patients with AF duration >12 months subgroup and age >60 years might experience greater benefits from AF acute termination with ablation. Importantly, acute termination of AF did not significantly increase the risk of operational complications.

Baker *et al*. [36] reviewed previous studies and found that the uninducible or termination of AF during ablation may indicate less structural remodeling, rather than serving as a definitive endpoint for the ablation. The meta-analysis of Li *et al*. [14] also suggests that the termination of AF is not a reliable endpoint in the ablation process for pers-AF. Our meta-study shows that termination of AF is associated with better clinical outcomes, regardless of the scope and strategy of ablation. A recent prospective, randomized, multicenter study has also confirmed that termination of AF during ablation is associated with a lower recurrence rate of AF [4]. While our study identifies acute AF termination as a strong predictor of long-term success, this association should not be conflated with causation. Instead, termination serves as a prognostic biomarker with several actionable clinical implications. Firstly, AF termination may act as a quality metric for ablation strategies. For instance, low termination rates in a center could prompt protocol reassessment (e.g., inadequate substrate modification, missed drivers) or adoption of advanced mapping tools. Secondly, it may help develop follow-up strategies. Patients without termination might benefit from closer surveillance (e.g., implantable loop recorders, quarterly Holter) to detect early recurrence and guide timely reintervention; Finally, termination may affect the use of antiarrhythmic drugs. Strengthen rhythm control in high-risk patients as early as possible, and termination could be tailored using termination status as a risk stratifier.

Electrical and structural remodeling are fundamental mechanisms that drive the progression of pers-AF. As the duration of AF increases, the heterogeneity of atrial substrate increases, which increases the susceptibility to pers-AF and the probability of maintaining AF. Most instances of AF can be terminated with PVI; however, additional ablative strategies, such as roof-line ablation and/or CFAE ablation, are often required to achieve successful termination of pers-AF, as supported by electrophysiological mapping findings. Moreover, the application of roof-line and/or CFAE ablation in patients with pers-AF not only facilitates acute AF termination but is also correlated with enhanced long-term clinical outcomes [37]. It is important to better describe and identify the potential “drivers” of AF, which may be suitable for ablation and improve treatment outcomes. The TARGET-AF1 trial [38] confirmed a close relationship between AF termination and sinus rhythm maintenance. As localized drivers are essential for AF maintenance, it appears that achieving ideal results of AF termination is contingent upon eliminating the maintenance mechanism of pers-AF. Linear ablation can also eliminate CFAE, which also confirms the effect of linear ablation on the modification of AF matrix in patients with pers-AF [29]. Researchers who oppose this endpoint argue that: termination may be difficult to achieve, and termination may only indicate the elimination of key drivers of AF during surgery but does not guarantee the elimination of all potential drivers of AF [39]. Researches indicate that artificial intelligence has been instrumental in achieving targeted, reproducible, and reliable identification of ablation targets. The application of AI-assisted ablation has significantly enhanced the one-year survival rate free from AF recurrence in patients with pers-AF, offering promising prospects for its further utilization in the field of AF ablation [40, 41].

Achieving acute AF termination during ablation is influenced not only by the aggressiveness of the ablation strategy but also by the inherent complexity of the atrial substrate [7, 42]. Pers-AF is maintained by a dynamic interplay of electrical drivers (e.g., rotors, focal sources) and structural remodeling (e.g., fibrosis, atrial dilatation) [42]. Patients with advanced substrate complexity—characterized by extensive fibrosis, larger left atrial diameters (>45 mm), or prolonged AF duration (>12 months)—often exhibit greater electrical heterogeneity and a higher burden of non-pulmonary vein (PV) drivers, making AF termination more challenging [43]. While stepwise ablation (PVI+substrate modification) increases termination rates compared to PVI alone, its success is substrate-dependent [7, 43]. In patients with localized drivers and minimal fibrosis, AF may terminate with limited ablation. Conversely, in diffusely remodeled atria, termination often requires extensive lesion sets, and even then, may not be achievable [6, 44]. These findings underscore the need for personalized ablation strategies that integrate pre-procedural assessment of substrate complexity (e.g., imaging, electro-anatomical mapping) to tailor lesion sets [44, 45].

Currently, AF duration greater than 12 months is classified as long-term pers-AF. According to the subgroup analysis of this study, patients with AF duration less than 12 months and greater than 12 months achieved favorable outcomes in terms of medium- and long-term success rates of AF, but for long-term pers-AF, acute termination of ablation may be more likely to predict a good clinical outcome. In addition, we also found that the acute termination of AF was more likely to predict a good clinical outcome in patients older than or less than 60 years old. Moreover, the tendency is more obvious in the age group older than 60 years old. We hypothesize that the following reasons may explain this result: Firstly, long-term pers-AF is associated with more extensive electrical and structural remodeling, including atrial fibrosis and conduction heterogeneity. While this may initially suggest a poorer prognosis, it also means that successful modification of the AF substrate during ablation (e.g., through linear ablation or complex fractionated electrogram ablation) can have a greater impact on restoring sinus rhythm [4]. Secondly, patients with more advanced fibrosis may respond better to extensive substrate modification, including additional linear ablations beyond PVI. Thirdly, in clinical practice, patients with longer-term AF and older age often receive more extensive ablation strategies, such as stepwise ablation with linear lesions and CFAE ablation. This may lead to higher termination rates and lower recurrence rates [29]. Lastly, aging is associated with increased sympathetic and vagal remodeling, which may affect AF maintenance and termination thresholds. Ablation strategies targeting autonomic modulation (e.g., ganglionated plexus ablation) may have a greater impact on older patients, improving long-term outcomes [46].

Our meta-analysis shows that the operation time in termination group has not increased, and the incidence of complications is similar to that of other ablation strategies. Since acute AF termination is not a universally adopted procedural endpoint, operators typically adhered to standardized ablation strategies irrespective of intraprocedural arrhythmia behavior. Thus, the absence of significant differences in complications underscores that pursuing AF termination does not inherently introduce additional risks when performed within established safety protocols.

The optimal ablation strategy of pers-AF is still a controversial challenge in clinical electrophysiology. Although a standardized provocation protocol for inducing non-PV triggers has not been established, the majority of electrophysiology laboratories recognize the critical role of non-PV triggers in AF. When such triggers are identified during ablation procedures, targeted ablation of these sites is considered essential [3]. It can’t be ignored that PV reconnection is still one of the main mechanisms of arrhythmia recurrence in most patients with recurrent AF [12]. Therefore, one of the current problems is the urgent need for a new technology to achieve transmural and lasting PVI through a single operation, and to minimize complications, to screen out those pers-AF patients who can benefit the most from PVI alone. We found a significant interaction was observed in the study design subgroup, where multi-center studies showed a success rate of RR 1.31 (95% CI, 1.14–1.50; *p* < 0.001), while single-center studies exhibited a higher success rate of RR 1.65 (95% CI, 1.49–1.82; *p* < 0.001), with an interaction *p*-value of 0.008. One potential explanation is that experienced centers achieve more consistent and stable outcomes with PVI and linear ablation techniques, likely due to refined procedural expertise and standardized protocols. However, with the advent of new technologies such as pulsed-field ablation and anhydrous alcohol ablation, the creation of linear blocks may be more secure. This advancement makes it easier to achieve the acute termination rate of AF and improves the long-term outcomes of persistent AF ablation.

## 5. Limitations

Firstly, the probability of acute termination of AF varies greatly in different studies. This variability is likely attributable to heterogeneity among patient populations, including differences in the presence of structural heart disease and the duration of atrial fibrillation AF. Such inconsistencies make it challenging to elucidate the benefits of achieving AF termination. Secondly, the ablation strategies employed in these studies vary significantly. The role of ablation methods and the duration of operation cannot be separated from the termination itself as an end point, and determining when to stop using these ablation protocols for ablation and declaring that AF cannot be terminated is always subjective. The variability in ablation strategies (e.g., PVI-only vs. stepwise substrate modification) represents a potential confounder. Aggressive substrate modification may increase acute termination rates but also raise the risk of iatrogenic atrial tachycardia. Future studies should stratify outcomes by ablation strategy to identify optimal approaches for specific patient subgroups. In addition, the current results are mostly based on retrospective studies, and more randomized controlled trials are needed to prove that ablation termination of AF is the end point of successful surgery. AS the quest for an optimal ablation procedure for pers-AF remains ongoing, the identification of a definitive procedural endpoint remains unresolved.

## 6. Conclusion

Our meta-analysis demonstrates that acute termination of AF during catheter ablation is significantly associated with improved long-term freedom from arrhythmia recurrence in patients with pers-AF. However, standardization of ablation strategies and randomized trials are needed to validate its role in improving outcomes for persistent AF.

## Availability of Data and Materials

The data that support the findings of this study are available from the corresponding author upon reasonable request.

## Supplementary Material

Supplementary material associated with this article can be found, in the online version, at https://doi.org/10.31083/RCM33419.
