# Supplementary material for: Atrial Fibrillation Termination Success During Ablation: Insights From Pooled Clinical Studies
Source: Rev Cardiovasc Med. 2025 Jul 30;26(7):33419. doi: 10.31083/RCM33419 (PMC12326452; doi:10.31083/RCM33419)
Supplement: Supplementary file 1 [file 2153-8174-26-7-33419-s1.zip › Supplementary Table+Figure.docx]

**Supplementary Table 1.** Baseline characteristics of the included populations

| **Characteristic** | **Overall (N=3,080)** | **Termination (N=1,655)** | **Non-Termination (N=1,425)** |
| --- | --- | --- | --- |
| Age (years),  mean ± SD | 59.2 ± 9.8 | 58.3 ± 9.1 | 60.2 ± 10.4 |
| Male, n (%) | 2,320 (75.3%) | 1,267 (76.5%) | 1,053 (78.2%) |
| Hypertension, n (%) | 1,680 (54.5%) | 860 (52%) | 820 (58%) |
| Diabetes Mellitus,  n (%) | 600 (19.5%) | 300 (18%) | 300 (22%) |
| LAD (mm),  mean ± SD | 44.5 ± 5.8 | 43.2 ± 5.0 | 46.1 ± 6.2 |

**Supplementary Table 2.** Subgroup analysis of freedom of AF between the termination group and non-termination group

| Subgroup Factors | Numbers of Study | RR (95%CI) | I^2^ (%) | *P* value | *P* for interaction |
| --- | --- | --- | --- | --- | --- |
| Study design |  | 1.31(1.14, 1.50)  1.65(1.49, 1.82)  1.58(1.39,1.79)  1.60 (1.41,1.81)  1.57(1.41,1.76)  1.63(1.41,1.89)  1.58(1.41,1.77)  1.90(1.48,2.45)  1.92(1.60,2.31)  1.51(1.33,1.70)  1.58(1.41,1.78)  1.85(1.49,2.29)  1.50(1.30,1.72)  1.92(1.47,2.50)  1.74(1.54,1.96)  1.48(1.22,1.79) |  |  | **0.008** |
| Multi-center | 2 |  | 0 | <0.001 |  |
| Single-center | 20 |  | 32.8 | <0.001 |  |
| Follow-up |  |  |  |  | 0.889 |
| >12 | 12 |  | 5.1 | <0.001 |  |
| ≤12 | 8 |  | 58.9 | <0.001 |  |
| Sample size |  |  |  |  | 0.690 |
| >50 | 12 |  | 52.8 | <0.001 |  |
| ≤50 | 9 |  | 0 | <0.001 |  |
| Male proportion |  |  |  |  | 0.185 |
| ≤80 | 13 |  | 47.5 | <0.001 |  |
| >80 | 5 |  | 0 | <0.001 |  |
| Age cutoff |  |  |  |  | **0.030** |
| >60 | 8 |  | 49.0 | <0.001 |  |
| ≤60 | 11 |  | 13.7 | <0.001 |  |
| HT proportion |  |  |  |  | 0.221 |
| <50 | 13 |  | 29.9 | <0.001 |  |
| ≥50 | 5 |  | 62.0 | <0.001 |  |
| LVEF |  |  |  |  | 0.105 |
| >55 | 10 |  | 0 | <0.001 |  |
| ≤55 | 4 |  | 0 | <0.001 |  |
| LAD(mm) |  |  |  |  | 0.163 |
| >40 | 15 |  | 16.6 | <0.001 |  |
| ≤40 | 2 |  | 0 | <0.001 |  |
| AF duration |  |  |  |  | **0.084** |
| >12 | 9 | 1.92(1.57,2.35) | 0 | <0.001 |  |
| ≤12 | 10 | 1.56(1.37,1.77) | 52.3 | <0.001 |  |
| DM proportion |  |  |  |  | 0.469 |
| >10 | 7 | 1.60(1.37,1.87) | 0 | <0.001 |  |
| ≤10 | 7 | 1.53(1.37,1.72) | 41.3 | <0.001 |  |

**Supplementary Fig. 1.** Forest plot of the long-term freedom from AF/ AFL/AT in random model


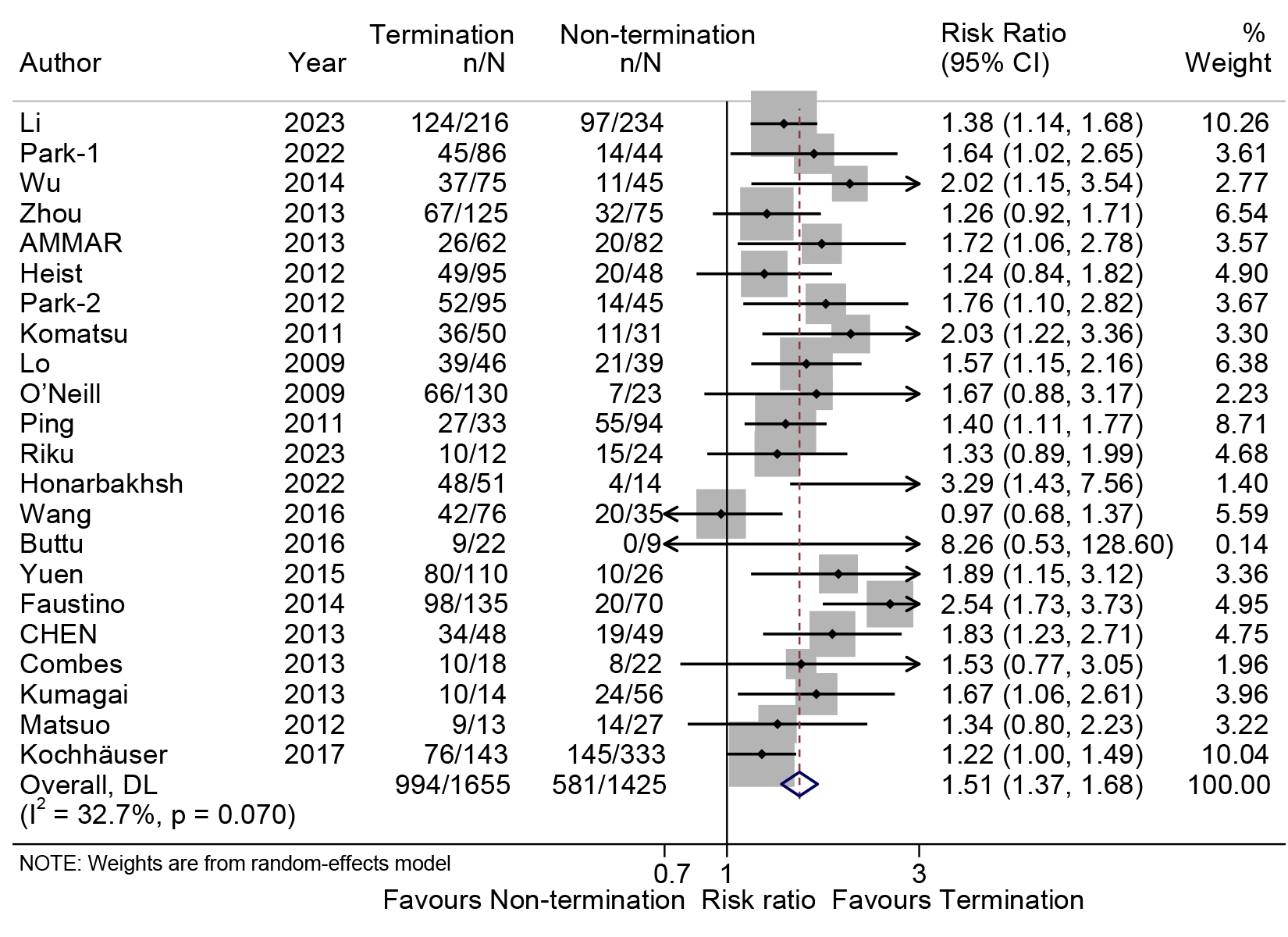


**Supplementary Fig. 2.** Risk of bias of included studies with trim and fill analysis in the meta-analysis


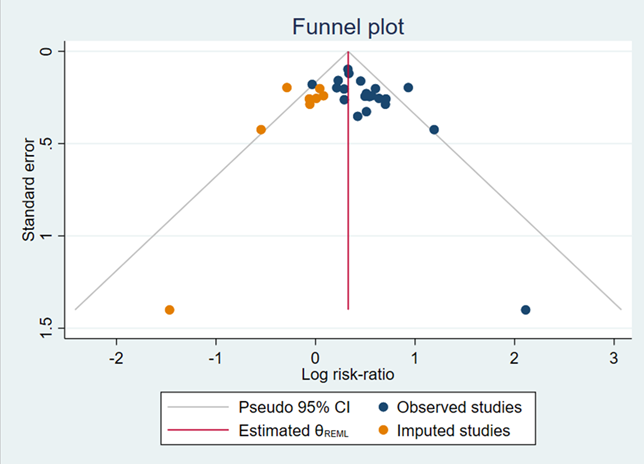


**Table 1. continued**

| First author, year, reference | Outcomes | follow-up duration(months) | follow-up strategies | Application of antiarrhythmic drugs |
| --- | --- | --- | --- | --- |
| Li-2023 | 57.4% of patients with termination of AF vs 41.5% without termination were AF free | 12 | Outpatient visits and 48-h Holter monitoring were scheduled at 1, 3, 6, 9, and 12 months, and every 6 months thereafter if the patient remained asymptomatic. Monthly telephone interviews were also done. All patients were asked to undergo additional ECG and 7-day Holter recordings when their symptoms were suggestive of tachycardia. | AAD (amiodarone, dronedarone, or propafenone) were prescribed for 1–2 months after ablation and were discontinued five half-lives before the end of the first 3 months (blanking period). |
| Riku-2023 | 83.3% of patients with termination of AF vs 62.5% without termination were AF free | 15.6 ± 6.3 | A 12-lead surface electrocardiogram was performed at every visit, and a 24-h Holter ECG was performed one month after ablation in all patients. Additional examinations and short follow-up visits were scheduled for patients with suspected symptom recurrences. | When patients had early recurrence within the blanking period, AAD that were discontinued before the procedure were re-administered. |
| Park-1-2022 | 52.3% of patients with termination of AF vs 31.8% without termination were AF free | 16±8 | Outpatient visits were made 1 week after discharge and then every 1 or 2 months thereafter. Standard ECG was performed at each visit, and the patient’s symptoms were checked. Holter monitoring was performed when symptoms were present; otherwise, the subjects underwent Holter monitoring every three months from the end of the blanking period | No mention |
| Honarbakhsh-2022 | 94.1% of patients with termination of AF vs 28.6% without termination were AF free | 29.5 ± 3.7 | All patients underwent clinical follow‐ up at 3, 6, and 12 months, with 48‐h ambulatory Holter monitoring at 6 and 12 months. Patients were followed up six monthly thereafter. | A 3‐month “blanking period” was observed, with all medication including AAD continued during this time. |
| Wang  -2016 | 55.3% of patients with termination of AF vs 57.1% without termination were AF free | 12 | After ablation, patients were followed up for 12 months. At each outpatient visit, a 12-lead ECG, 24 hours Holter, and echocardiographic study were performed. | No mention |
| Buttu  -2016 | 40.9% of patients with termination of AF | 35±15 | Clinical follow-up was performed at scheduled visits at 3, 6, 12, 18, and 24 months, then every year, and included echocardiographic evaluation, ECG, and 48-h Holter recordings. | No mention |
| Yuen  -2015 | 72.7% of patients with termination of AF vs 38.5% without termination were AF | 15±7 | Patients were followed up at outpatient clinics 1 or 2 weeks after discharge, then every 2 – 4 weeks during the first 3 months (blanking period) to monitor early recurrence of arrhythmia. They were seen every 3 months after the blanking period.In asymptomatic patients, 24 h Holter monitoring was performed at 3, 6, 9, and 12 months after discharge. Symptomatic patients were asked to report to our clinic early and loop recorder would be considered if ECG and 24 h Holter monitoring could not demonstrate any arrhythmia recurrence. | AAD were given in all patients in the blanking period and stopped after blanking period if no early recurrence of arrhythmia was observed. For patients with early recurrence, whether antiarrhythmic drugs were continued after blanking period was at the discretion of the physicians. In patients with AF or AT occurring during follow-up, AAD were given at the discretion of the physicians. |
| Wu-2014 | 49.3% of patients with termination of AF vs 24.4% without termination were AF | 61.2±13.2 | An ECG and 24-hour Holter monitor recording were obtained before discharge and repeated after 1, 3, 6, and 12 months. Then a clinical follow-up was regularly carried out every 6 months, which included performing an ECG and 24-hour Holter monitor recording. | The AAD were continued for 3 months after the procedure, and then the AAD was stopped if no AF recurrence was recorded or reported. |
| Faustino  -2014 | 72.6% of patients with termination of AF vs 28.6% without termination were AF | 12 | After catheter ablation, all patients were followed in our outpatient clinic every 3, 6 and 12 months, and data were collected (a) using a 12-lead ECG and a 24-hour Holter monitor; (b) using a structured questionnaire on arrhythmia recurrence and any other symptom. | No mention |
| CHEN  -2013 | 70.8% of patients with termination of AF vs 38.8% without termination were AF | 12.0 ± 9.5 | Patients were followed at outpatient clinics 1 or 2 weeks after discharge then every 2–4 weeks during the first 3 months (blanking period) to monitor the early recurrence of arrhythmia and also the occurrence of possible complications, for example, delayed tamponade, sick sinus syndrome, phrenic nerve injury, or atrio-esophageal fistula formation, etc., and every 3 months after the blanking period. ECG was routinely performed during each time outpatient clinics follow-up. 24-hour Holter monitoring was performed at 3, 6, 9, and 12 months after discharge in asymptomatic patients and loop recorders used for symptomatic patients. | AAD were given in all patients in the blanking period. In patients with AF or AT occurring during follow-up, antiarrhythmic drugs were given at the discretion of the physicians. |
| Zhou  -2013 | 53.6% of patients with termination of AF vs 42.7% without termination were AF | 50.0 ± 9.3 | Patients were asked to schedule an outpatient visit once a month for 1 year and then every 6 months or at any time the patient had tachycardia-related symptoms. Then 24-hour ambulatory monitoring was performed 1, 3, 6 months after the procedure, and at least once every 6 months thereafter for at least 36 months. | The AADs amiodarone or propafenone were administered on the day of the procedure if not contraindicated. Withdrawal of amiodarone and propafenone was attempted if patients were in stable SR 1–3 months after the procedure. |
| Combes  -2013 | 55.6% of patients with termination of AF vs 36.4% without termination were AF | 12 | Patients were assessed before discharge and at the third, sixth and 12th months by clinical interview, echocardiography and 24-h Holter monitoring. In addition, patients were instructed to call their cardiologist in case of sustained palpitation, for immediate ECG recording. | Amiodarone was continued for at least 3 months in patients who were receiving amiodarone before the procedure and was interrupted in case of no recurrence at 3 months. |
| Kumagai  -2013 | 71.4% of patients with termination of AF vs 42.9% without termination were AF | 12±4.1 | Clinical interview, surface ECG or 24-h Holter monitoring were performed 1 day after the procedure and repeated 1, 3, 6 and 12 months thereafter by the referring cardiologist.When the patients had any clinical symptomatic palpitations after the AF ablation, examinations including ECG, 24-h Holter monitoring and assessment of the current condition were also performed on an outpatient basis. | The AAD were continued for at least 3 months to prevent any early recurrences of AF. |
| AMMAR-2013 | 41.9% of patients with termination of AF vs 24.4% without termination were AF | 12 | Patients history from 3-monthly outpatient clinic visits and repeated 7-days-Holter-ECGs (every 3 months and in case of symptoms) were used to assess arrhythmia burden during 12 months after ablation. | No mention |
| Matsuo  -2012 | 69.2% of patients with termination of AF vs 51.9% without termination were AF | 19.7±3.3 | The presence/absence of atrial tachyarrhythmia was evaluated by their symptoms, ECG recordings, and 24-hour ambulatory monitoring (1 month and every 3 months after the procedure). All patients who had symptoms without documentation of AF recurrence were given an event recorder (used for 5 days) to identify the cause of their symptoms. | No mention |
| Heist  -2012 | 51.6% of patients with termination of AF vs 41.7% without termination were AF | 33 | The patients were followed up in the office at regularly scheduled visits, generally 4 to 6 weeks after ablation and 3, 6, and 12 months after ablation, and yearly thereafter, with additional visits scheduled if symptoms suggestive of arrhythmia recurrence were noted. Recurrence of arrhythmia (symptomatic or asymptomatic) was determined from electrocardiograms performed during routine office visits (and at urgent visits for symptomatic arrhythmias) and outpatient Holter monitoring or multiweek event monitoring using event monitors with auto-triggering for AF, AT, and other atrial arrhythmias | AAD were continued at the discretion of the operator for 1 to 2 months after the ablation procedure. The AAD were then discontinued for patients free of arrhythmia after this period. |
| Park-2-2012 | 54.7% of patients with termination of AF vs 31.1% without termination were AF | 18.7 ± 7.6 | The patients were seen in an outpatient clinic at 1 week, 1, 3, 6, 9, and 12 months after the procedure and then every 6 months thereafter. A 12-lead surface electrocardiogram was performed at every visit. Patients were evaluated by 24- or 48-hour holter monitoring or 7-day event recorder at 3, 6, 9, 12, 18, 24, 30, and 36 months after the ablation. | At the 3-month visit, AAD were discontinued if there was no evidence of recurrence. |
| Komatsu-2011 | 72.0% of patients with termination of AF vs 35.5% without termination were AF | 14±7 | The first outpatient clinic visit was 3 weeks after the ablation procedure. The patients then underwent follow-up consisting of clinical interview, ECG, and 24h Holter monitoring every 1– 2 months at our cardiology clinic. If any symptoms suggestive of an arrhythmia occurred between scheduled visits, the patients were asked to come to the emergency department, and ECG, 24 h Holter monitoring, and/or cardiac event recording with a recording duration of 1 month were performed to define the cause of the symptoms. | All AADwere discontinued within 2 months after ablation. |
| Lo  -2009 | 84.8% of patients with termination of AF vs 53.8% without termination were AF | 13± 8 | After discharge, patients underwent follow-up (2 weeks after catheter ablation, then every 1–3 months thereafter) at our cardiology clinic or with the referring physician. Long-term efficacy was assessed clinically on the basis of clinical symptoms, resting surface 12-lead ECG, and 24-hour Holter monitoring and/or 1-week cardiac event recorder recordings. | AAD were prescribed for 8 weeks to prevent any early recurrence of AF (defined as less than 2 months after ablation) If more than one episode of atrial flutter, recurrent symptomatic AF, or atrial tachycardia was documented, patients were encouraged to undergo another ablation procedure, or AAD were prescribed to control the recurrent atrial arrhythmias. |
| O’Neill  -2009 | 50.8% of patients with termination of AF vs 30.4% without termination were AF | 34 ± 3 | Patients were routinely hospitalized for up to 5 days post-procedure and again for 1 day at 1, 3, 6, and 12 months for clinical interview and ambulatory monitoring in addition to later routine follow-up by the referring cardiologist including Holter monitoring in the event of symptoms. | AAD was continued for 1–3 months following the index procedure. |
| Ping-2011 | 81.8% of patients with termination of AF vs 58.5% without termination were AF | 28±7 | Follow—up analyses were conducted for all symptomatic patients using 12- lead ECG and 24．hour Holter monitoring directly before discharge as well as at 1，3，6，and 12 months after ablation. | Unless contraindicated．all patients received AAD (amiodarone or propafenone)for three months after ablation. |
| Kochhäuser-2017 | 53.1% of patients with termination of AF vs 43.5% without termination were AF | 18 | An initial blanking period of 3 months was used and follow-up visits along with 24 hour Holters were performed 3, 6, 9, 12 and 18 months after the initial ablation procedure. Weekly transtelephonic monitoring (TTM) was also performed, with additional recordings in the events of symptoms. | AAD (except amiodarone) was allowed during the first 3 months after initial ablation. After 3 months, antiarrhythmic therapy had to be stopped to assess for recurrence of AF. |
